# Supplementary material for: Unique, Specific CART Receptor-Independent Regulatory Mechanism of CART(55-102) Peptide in Spinal Nociceptive Transmission and Its Relation to Dipeptidyl-Peptidase 4 (DDP4)
Source: Int J Mol Sci. 2023 Jan 4;24(2):918. doi: 10.3390/ijms24020918 (PMC9865214; doi:10.3390/ijms24020918)

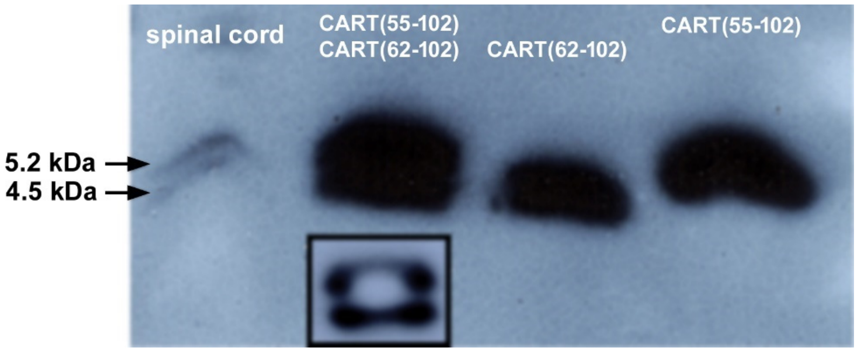

Supplementary Figure S1. Western blot analysis confirmed existence of CART peptide fragments in the rat spinal cord. Both CART(55-102) and CART(62-102) peptide fragments could be detected in the rat spinal cord. Insert represents the doublet of the CART(55-102) + CART(62-102) lane from a separate run with shorter exposure. Photos of the original Wester blot gels from which the upper image was constructed are seen below.

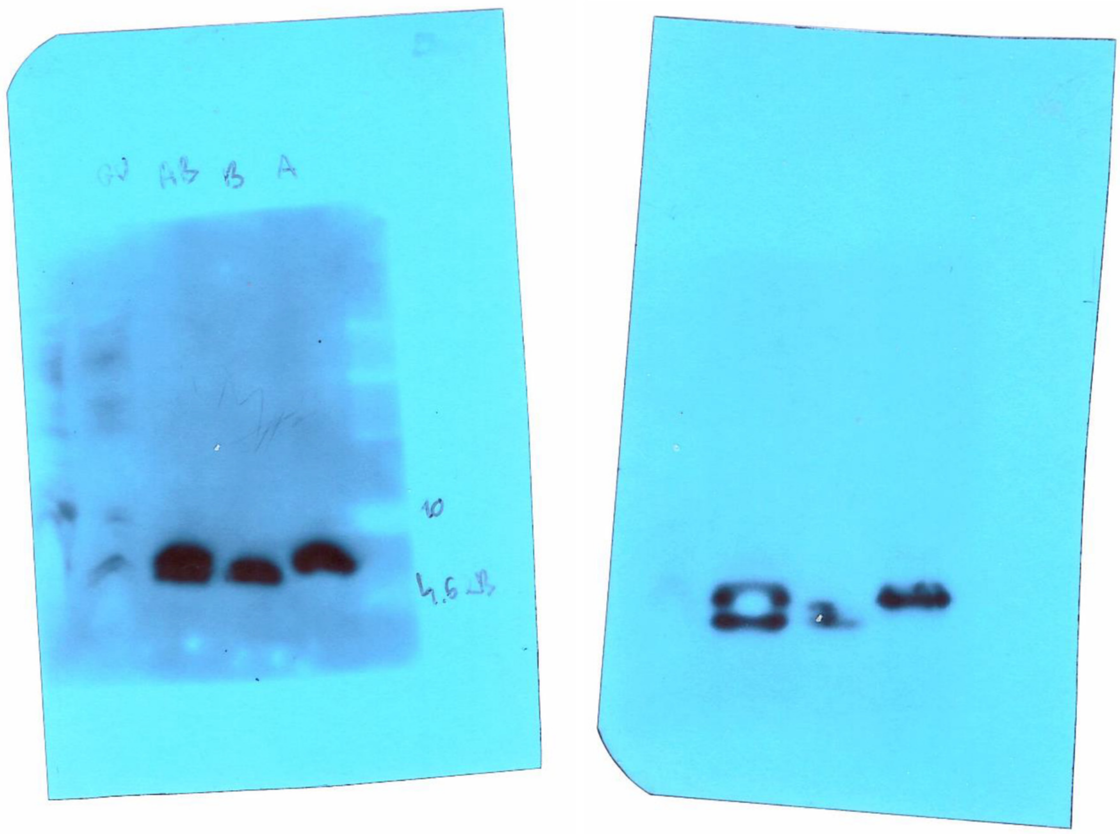

Supplement: Supplementary file 1 [file ijms-24-00918-s001.zip › Supplementary Figure S1.pdf]
