# Supplementary material for: Unique, Specific CART Receptor-Independent Regulatory Mechanism of CART(55-102) Peptide in Spinal Nociceptive Transmission and Its Relation to Dipeptidyl-Peptidase 4 (DDP4)
Source: Int J Mol Sci. 2023 Jan 4;24(2):918. doi: 10.3390/ijms24020918 (PMC9865214; doi:10.3390/ijms24020918)
Supplement: Supplementary file 1 [file ijms-24-00918-s001.zip › Supplementary Figure S2.pdf]

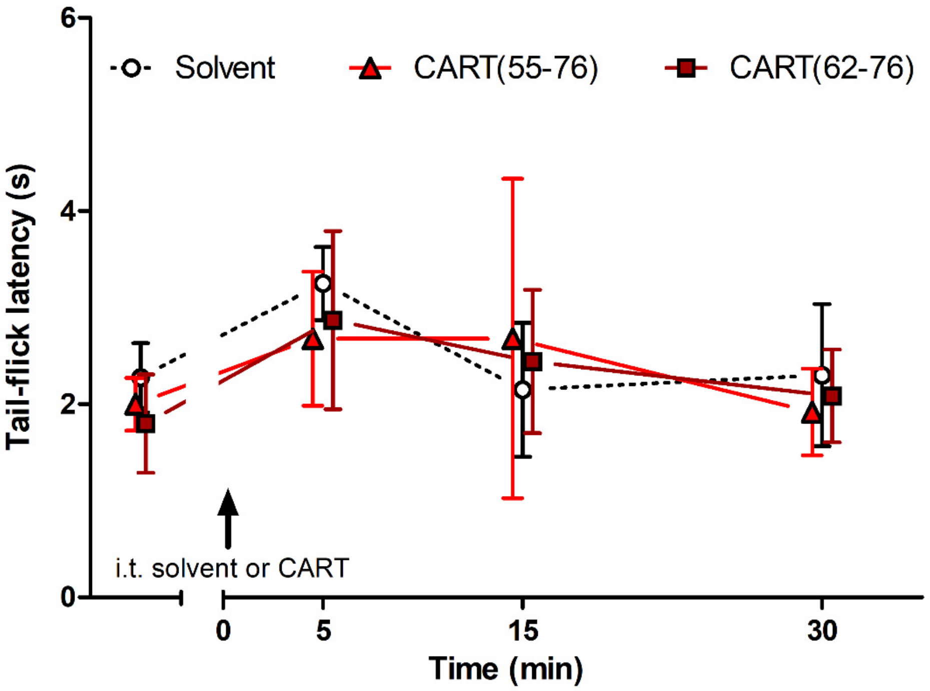

Supplementary Figure S2. CART fragments have no effect on tail-flick latency. The tail-flick latency was measured once before, and 5, 15 and 30 minutes after intrathecal (i.t.) drug application. CART(55-76) and CART(62-76) were tested on 5 and 7 rats, respectively, and 6 animals were used as controls. Neither the CART(55-76), nor the CART(62-76) applied in 10 nmol/rat concentrations altered the acute nociceptive thresholds significantly. (Two-way repeated measures ANOVA with Bonferroni post hoc test, $p > 0.05$). Data are given as mean ± SD.
