# Supplementary material for: Unique, Specific CART Receptor-Independent Regulatory Mechanism of CART(55-102) Peptide in Spinal Nociceptive Transmission and Its Relation to Dipeptidyl-Peptidase 4 (DDP4)
Source: Int J Mol Sci. 2023 Jan 4;24(2):918. doi: 10.3390/ijms24020918 (PMC9865214; doi:10.3390/ijms24020918)
Supplement: Supplementary file 1 [file ijms-24-00918-s001.zip › Supplementary Figure S3.pdf]

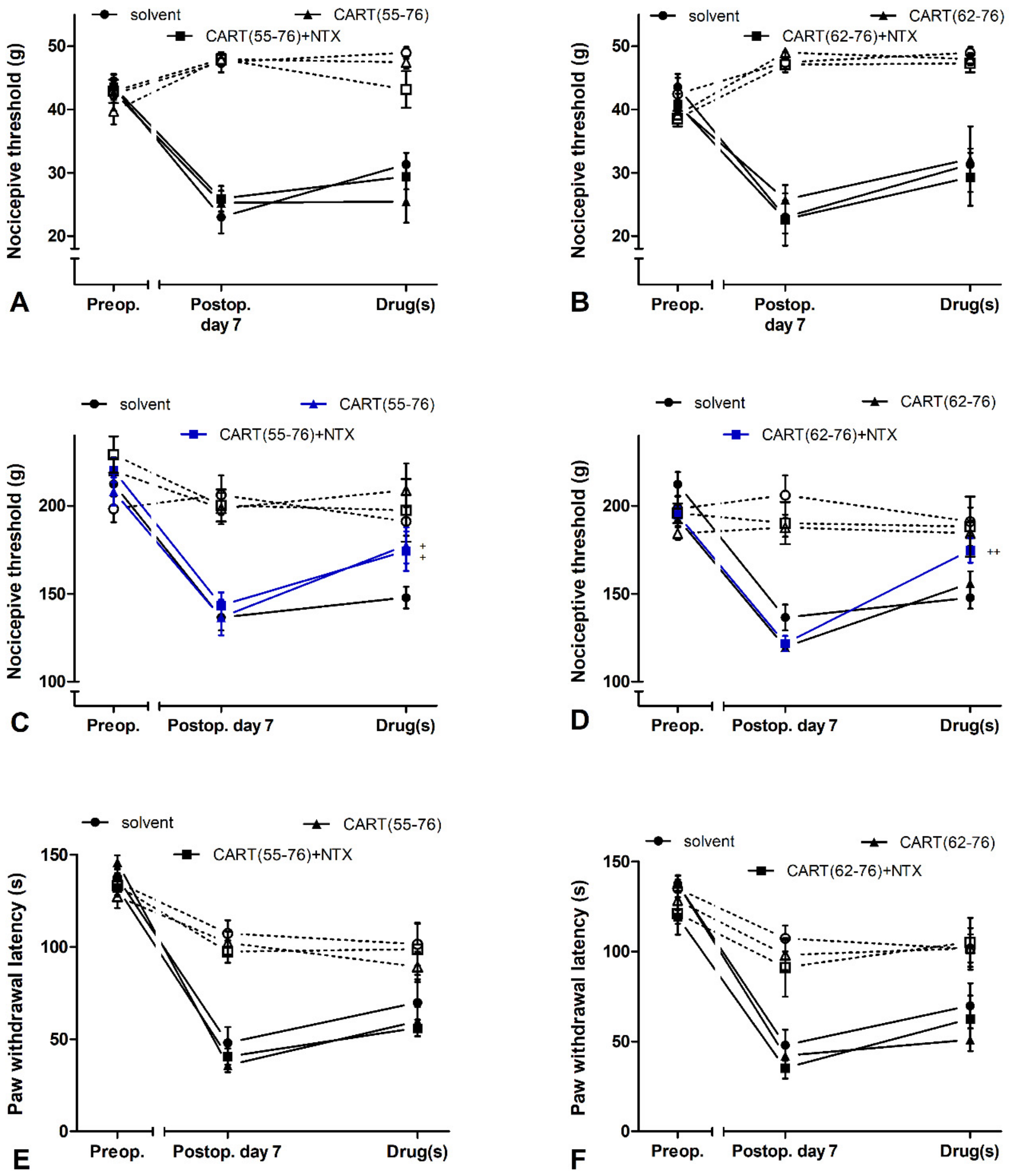

Supplementary Figure S3. Antinociceptive effects of CART(55-76) and CART(62-76) fragments in chronic neuropathic pain related to partial sciatic nerve ligation. Both peptides were ineffective on neuropathic mechanical allodynia (**A**,**B**) and cold hyperalgesia (**E**,**F**) tests. However, CART(55-76) (3 nmol/rat, i.t.) had an inhibitory action on mechanical hyperalgesia, which was not antagonized by NTX (0.5mg/g b.w., s.c.) (**C**). Surprisingly, CART(62-76) (10 nmol/rat, i.t.) was ineffective alone but when was co-administered with NTX, an antihyperalgesic effect was observed (**D**). Altogether 41 rats developing a minimum of 20% decrease of nociceptive thresholds following partial sciatic nerve ligation were used in this set of experiments. Comparisons were made with two-way ANOVA, Bonferroni post hoc test; +: $p < 0.05$; ++: $p < 0.01$. Crosses indicate significant difference between the time-matching points of CART fragments and CART peptides + NTX curves. Filled symbols: side of ligature; open symbols with dashed lines: contralateral side. Data on each curves and bars are given as mean and SEM.
