# Supplementary material for: Unique, Specific CART Receptor-Independent Regulatory Mechanism of CART(55-102) Peptide in Spinal Nociceptive Transmission and Its Relation to Dipeptidyl-Peptidase 4 (DDP4)
Source: Int J Mol Sci. 2023 Jan 4;24(2):918. doi: 10.3390/ijms24020918 (PMC9865214; doi:10.3390/ijms24020918)
Supplement: Supplementary file 1 [file ijms-24-00918-s001.zip › Supplementary Figure S4.pdf]

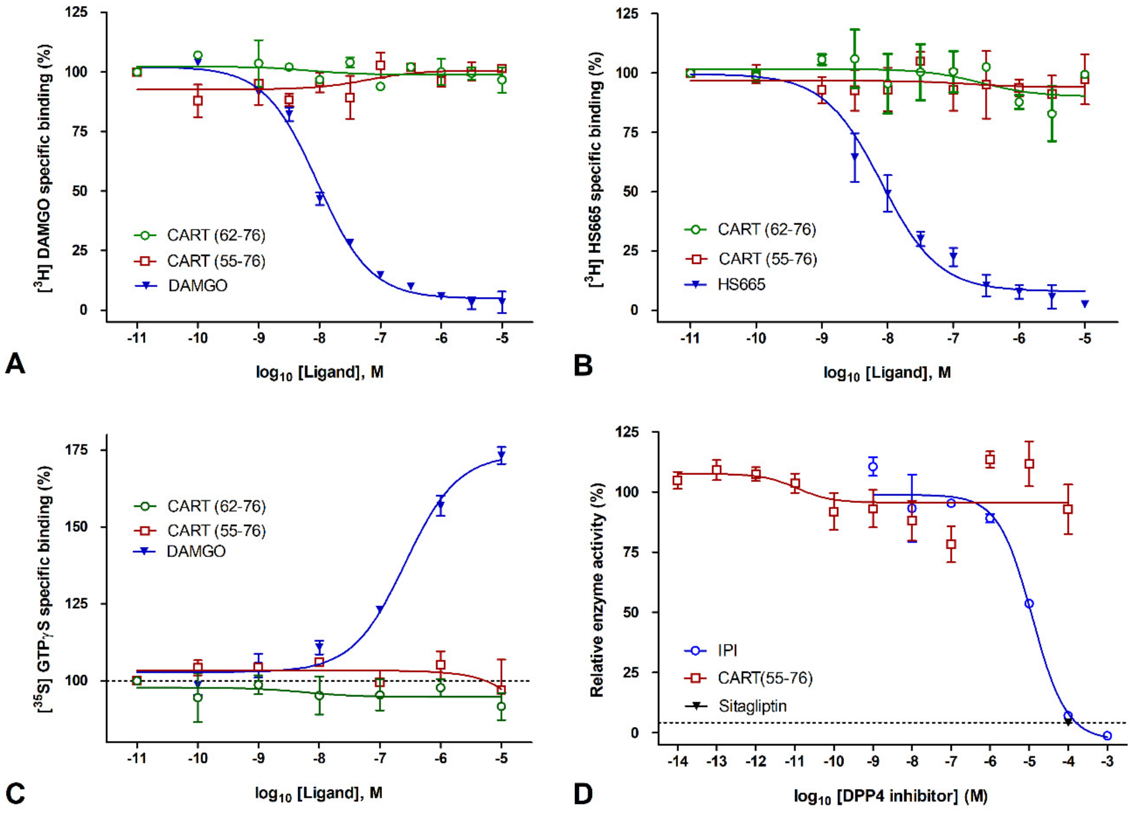

Supplementary Figure S4. CART fragments do not bind to any types of opioid or G protein-coupled receptors and the complete CART(55-76) is not acting as a DPP4 inhibitor. MOR (**A**) and KOR (**B**) binding affinity of CART peptides to DAMGO and HS665 in $[^3H]$DAMGO and $[^3H]$HS665 competition binding assays in rat and guinea pig brain membrane homogenates, respectively. Values represent mean values±SEM for at least three experiments performed in duplicate. Specific binding of $[^{35}S]$GTPγS in percentage (**C**) in the presence of increasing concentrations (10^{-10} - 10^{-5} M) of the indicated ligands. In the enzyme inhibitory assay (**D**) the level of basal activity was defined as 100% and maximal inhibitory effect of sitagliptin was indicated with a dotted line. Points represent means±SEM for at least three experiments performed in triplicates.
