# Supplementary material for: Unique, Specific CART Receptor-Independent Regulatory Mechanism of CART(55-102) Peptide in Spinal Nociceptive Transmission and Its Relation to Dipeptidyl-Peptidase 4 (DDP4)
Source: Int J Mol Sci. 2023 Jan 4;24(2):918. doi: 10.3390/ijms24020918 (PMC9865214; doi:10.3390/ijms24020918)
Supplement: Supplementary file 1 [file ijms-24-00918-s001.zip › Supplementary Table S1.pdf]

Supplementary Table S1. Primary and fluorescent secondary antibodies used for immunohistochemistry and ELISA

Primary Antibodies	Species	Dilution	Source	Catalog No	Characterization/ Specificity
CART	mouse	1:1500	gift from J. T. Clausen, Novo Nordisk, Denmark	-	[7]
CGRP	guinea pig	1:5000	Bachem (Peninsula Laboratories)	T-5027	[19,79]
CAV1	rabbit	1:500	Cell Signalling	3267	[96]
DPP4	goat	1:500	Novus Biological (Bio-Techne)	NB100-61658	[33]
GFAP	guinea pig	1:2000	Synaptic Sytems	173 004	[97]
IL-6	rabbit	1:500	PeproTech	500-P65	[98]
SP	rat	1:500	Bio-Rad (AbD Serotec)	8450-0505	[80]
TLR4	mouse	1:1000	Abcam	ab22048	[95]
TNFα	rabbit	1:500	PeproTech	500-P64	[100]
**Secondary Antibodies**	**Species**	**Dilution**	**Source**	**Catalog No**	
anti-mouse Alexa 488	donkey	1:2000	Invitrogen (Thermo Fisher Scientific)	A32766	
anti-mouse-Alexa 555	donkey	1:500	Invitrogen (Thermo Fisher Scientific)	A-31570	
anti-rat Cy5	donkey	1:100	Jackson Immunoresearch	712-175-150	
anti-guinea pig Alexa 648	donkey	1:2000	Invitrogen (Thermo Fisher Scientific)	A21450	
anti-guinea pig Cy5	donkey	1:100	Jackson Immunoresearch	706-175-148	
anti-rabbit Alexa 555	donkey	1:2000	Invitrogen (Thermo Fisher Scientific)	A32794	
anti-goat-Alexa 488	donkey	1:500	Invitrogen (Thermo Fisher Scientific)	A-11055	
anti-goat Alexa 555	donkey	1:2000	Invitrogen (Thermo Fisher Scientific)	A21432	
